# Risk of Comorbid Insomnia Disorder Associated with Major Depression in Apneic Patients: A Cross-Sectional Study

**DOI:** 10.3390/clockssleep6030026

**Published:** 2024-07-26

**Authors:** Matthieu Hein, Benjamin Wacquier, Matteo Conenna, Jean-Pol Lanquart, Camille Point

**Affiliations:** 1 Hôpital Universitaire de Bruxelles, Service de Psychiatrie et Laboratoire du Sommeil, Université Libre de Bruxelles, ULB, 1070 Brussels, Belgium; benjamin.wacquier@hubruxelles.be (B.W.); matteo.conenna@hubruxelles.be (M.C.); secmed.psy.erasme@hubruxelles.be (J.-P.L.); camille.point@hubruxelles.be (C.P.); 2 Laboratoire de Psychologie Médicale et Addictologie (ULB312), Université Libre de Bruxelles, ULB, 1020 Brussels, Belgium

**Keywords:** insomnia disorder, major depression, obstructive sleep apnea syndrome, polysomnography, risk factor

## Abstract

Given the limitations of available studies, the objective of this study was to explore the role played by current and remitted major depression in the occurrence of comorbid insomnia disorder for apneic patients. Data from 1488 apneic patients were extracted from the medical reports of polysomnographic recordings available in the database of the Sleep Laboratory. The presence of comorbid insomnia disorder in these apneic patients was defined based on the diagnostic criteria of the American Academy of Sleep Medicine Work Group. The risk of comorbid insomnia disorder associated with current or remitted major depression in apneic patients was investigated using multivariate logistic regression models. After adjustment for the main confounding factors, multivariate logistic regression analyses revealed that remitted and current major depression were significantly associated with the occurrence of comorbid insomnia disorder in apneic patients. The findings of this study seem to indicate that comorbid insomnia disorder could be a residual symptom and a marker of major depression in apneic patients, which justifies the establishment of an adequate treatment for major depressive episodes and their potential residual symptoms to allow the better management of comorbid insomnia disorder and the better prevention of its potential negative consequences in this particular subpopulation.

## 1. Introduction

In the literature, there are arguments in favor of a particular relationship between obstructive sleep apnea syndrome (OSAS) and insomnia disorder. Indeed, insomnia disorder is a frequent comorbidity (43.0%) in apneic patients, whereas the prevalence of OSAS is estimated at 34.5% among insomniac patients [1,2]. Furthermore, in apneic patients, the occurrence of comorbid insomnia disorder seems to favor the development of cardiovascular diseases since apneic patients with comorbid insomnia disorder have a higher risk of cardiovascular disease than those without comorbid insomnia [3,4,5]. In addition, the presence of comorbid insomnia disorder appears to be associated with a negative impact on mental health, life quality and professional performance in apneic patients [6]. Concerning the therapeutic aspect, it has been shown that apneic patients with comorbid insomnia disorder are less adherent and compliant to OSA treatments than those without comorbid insomnia disorder [7,8]. Thus, given these data, it seems essential to identify the potential factors favoring the occurrence of comorbid insomnia disorder in apneic patients in order to allow the better management of this sleep disorder in this particular subpopulation.

Based on the available studies, some evidence seems to indicate the existence of a bidirectional relationship between major depression and insomnia disorder. Indeed, insomnia complaints are very frequent among major depressed patients and the prevalence of depressive symptoms is high among insomniac patients [9,10]. In addition, insomnia disorder is associated with an increased risk of developing a major depressive episode, whereas major depression may lead to the occurrence of insomnia complaints [11,12]. Furthermore, in major depressed patients in remission, insomnia complaints are a frequent residual symptom that may contribute to the occurrence of depressive relapse [13,14]. However, despite the high prevalence of major depression in apneic patients [15], few studies have currently investigated the role played by this psychiatric disorder in the occurrence of comorbid insomnia disorder for this particular subpopulation [16,17,18,19,20]. In addition, most of these studies have mainly investigated the impact of depressive symptoms, measured by a self-questionnaire or a self-reported diagnosis of depression, on the occurrence of insomnia complaints in apneic patients, which may limit the interpretation of their results [16,17,18,19,20]. In this context, it could therefore be interesting to study the risk of comorbid insomnia disorder associated with remitted and current major depression (diagnosed during a systematic psychiatric interview) in apneic patients to allow the better identification of individuals at risk of insomnia complaints in this particular subpopulation.

Given these different previous data available in the literature, the hypothesis of this study was that remitted and current major depression are associated with an increased risk of comorbid insomnia disorder in apneic patients, which could indicate that this sleep disorder is a residual symptom and a marker of major depression in this particular subpopulation. To confirm this hypothesis, the risk of comorbid insomnia disorder associated with remitted and current major depression was investigated in a large sample of apneic patients. The goal of this approach is to allow health professionals to obtain reliable data regarding the risk of comorbid insomnia disorder associated with remitted and current major depression in apneic patients in order to allow the better prevention of the multiple negative consequences associated with the occurrence of this sleep disorder in this particular subpopulation.

## 2. Results

### 2.1. Polysomnographic Data (Table 1)

Apneic patients with comorbid insomnia disorder had a longer sleep latency than those without comorbid insomnia disorder. In addition, the sleep efficiency, sleep period time, micro-arousal index, apnea–hypopnea index, oxygen desaturation index and total time under 90% of SaO_2_ were lower in apneic patients with comorbid insomnia disorder than in those without comorbid insomnia disorder. The two groups of apneic patients did not differ significantly for other polysomnographic parameters.

### 2.2. Demographic Data (Table 2)

Insomnia disorder was a frequent comorbidity (40.9%) in our sample of apneic patients. Sex, age, body mass index, antidepressant therapy, benzodiazepine receptor agonists, cardiometabolic comorbidities, OSAS severity, sleep duration, excessive daytime sleepiness and the major depression status were significantly associated with the occurrence of comorbid insomnia disorder in apneic patients. Furthermore, compared to those without comorbid insomnia disorder, apneic patients with comorbid insomnia disorder were younger in age and had higher scores on the Beck Depression Inventory/Insomnia Severity Index/Epworth Sleepiness Scale. The two groups of apneic patients did not differ significantly for other demographic parameters. Finally, the prevalence of remitted and current major depression was 16.6% and 23.9%, respectively, in our sample of apneic patients.

### 2.3. Multivariate Analyses (Table 3)

After adjustment via the hierarchical introduction of the main confounding factors identified during the univariate analyses, the multivariate logistic regression analyses revealed that remitted and current major depression were significantly associated with the occurrence of comorbid insomnia disorder in apneic patients.

## 3. Discussion

Given the high prevalence of comorbid insomnia disorder (40.9%) in our sample of apneic patients, the results of our study seem to confirm that this sleep disorder is a frequent comorbidity in this particular subpopulation [3]. However, this prevalence is higher than that in the studies by Stelzer et al. (2021) (29.0%) and Cho et al. (2018) (29.2%), which could be explained by the recruitment of more severe apneic patients in these studies than in our study [17,21]. Indeed, in the literature, it has been shown that insomnia complaints tend to decrease with OSAS severity [22,23], which may have led to an underestimation of the prevalence of comorbid insomnia disorder in these two studies. Furthermore, the prevalence of comorbid insomnia disorder highlighted in our study is lower than that of the studies by Wallace et al. (2019) (74%) and Hagen et al. (2009) (61%), which could be explained by the fact that unlike our study where the comorbid insomnia disorder was diagnosed based on the American Academy of Sleep Medicine Work Group diagnostic criteria, insomnia complaints were investigated in these two studies using self-questionnaires [18,20]. However, it has been shown that the use of self-questionnaires for the diagnosis of insomnia disorder may promote the overdiagnosis of this sleep disorder [24], which could provide a better understanding of this higher prevalence of insomnia complaints in these two latter studies. Finally, the prevalence of comorbid insomnia disorder demonstrated in our study is similar to that of the meta-analysis by Zhang et al. (2019) (38.0%), which seems to confirm that despite the methodological differences between the available studies, apneic patients are a subpopulation at high risk of comorbid insomnia disorder [25]. Thus, in this context, it seems essential to systematically search for the presence of comorbid insomnia disorder in apneic patients given the potential negative consequences associated with this sleep disorder in this particular subpopulation.

Similar to the available literature [3,15,26], we demonstrated that remitted and current major depression are frequent comorbidities in apneic patients since their prevalence was, respectively, 16.6% and 23.9% in our sample, which confirms that the occurrence of this psychiatric disorder is a significant problem in this particular subpopulation. In addition, we have shown that remitted and current major depression are significantly associated with the occurrence of comorbid insomnia disorder in apneic patients. However, this high prevalence of remitted and current major depression and their potential implication in the occurrence of comorbid insomnia disorder for apneic patients may potentially be explained by several pathophysiological elements. First, excessive sleep fragmentation and intermittent hypoxia related to OSAS may induce biological alterations (modification of cerebral monoaminergic neurotransmission, activation of pro-inflammatory mechanisms and alteration of some cerebral structures [hippocampus and frontal lobes]) and promote the occurrence of complaints of excessive daytime sleepiness in apneic patients [27,28,29]. However, there are numerous arguments in favor of the central involvement of these biological alterations and complaints of excessive daytime sleepiness in the pathophysiology of major depression, which could provide a better understanding of the high prevalence of this psychiatric disorder highlighted in our sample of apneic patients [27,28,29]. Second, in patients with current major depression, one of the theories currently proposed to explain the frequent occurrence of insomnia disorder is the phenomenon of hyperarousal that may be divided into three highly interrelated categories: somatic, cortical and cognitive hyperarousal [30,31,32,33]. The presence of hyperarousal in patients with current major depression is characterized by the existence of a state of hypervigilance present throughout the 24-hour cycle, favoring the occurrence of complaints of insomnia (difficulty falling asleep, nocturnal awakenings and early morning awakenings) [30,31,32,33]. The occurrence of hyperarousal related to current major depression could be one of the main pathophysiological mechanisms related to the increased risk of comorbid insomnia disorder associated with this psychiatric disorder in the apneic patients in our sample. Third, in patients with remitted major depression, the phenomenon of hyperarousal may persist despite the remission of the main depressive symptoms [34,35,36]. However, this persistence of the phenomenon of hyperarousal in patients with remitted major depression may be manifested by the existence of a residual insomnia disorder favoring the occurrence of depressive relapses [34,35,36], which could help to better understand the increased risk of comorbid insomnia disorder associated with remitted major depression in our sample of apneic patients. Thus, these different elements seem to indicate that comorbid insomnia disorder could be a residual symptom and a marker of major depression in apneic patients, which seems to justify systematic screening for this psychiatric disorder in patients with OSAS and comorbid insomnia disorder.

The demonstration of this increased risk of comorbid insomnia disorder associated with current or remitted major depression in apneic patients could open up new therapeutic perspectives for the management of this sleep disorder in this particular subpopulation. Indeed, given that insomnia complaints are one of the symptoms frequently present during major depressive episodes [37,38], it is important to start an appropriate antidepressant treatment in apneic patients with current major depression in order to target the complete remission of the affective, cognitive and neurovegetative symptoms of this psychiatric disorder [39,40]. However, even in the case of clinical remission, the optimization of this antidepressant treatment may be necessary in some apneic patients with remitted major depression to avoid the persistence of residual insomnia complaints [39,40]. Regarding psychotherapeutic treatments that could be used alone or in combination with antidepressant treatment in apneic patients with current or remitted major depression, cognitive–behavioral therapy for insomnia seems to be a promising option given its positive results for both depressive symptoms and insomnia complaints [41]. Indeed, it has been demonstrated that cognitive behavioral therapy for insomnia may be used to enhance the effectiveness of antidepressant treatments in patients with current major depression and treat residual insomnia complaints in patients with remitted major depression [13,42]. Furthermore, alongside this specific management of major depression in apneic patients, it is essential to adequately treat OSAS in order to reinforce the improvement of depressive symptoms and to avoid the persistence of pathophysiological mechanisms related to obstructive respiratory events that may promote the maintenance of insomnia complaints [43,44]. Thus, in patients with OSAS and major depression, the establishment of an adequate combined treatment of these two pathologies could allow the better management of comorbid insomnia disorder and the better prevention of its potential negative consequences.

Finally, although there is evidence showing that more frequent alcohol consumption and smoking are associated with insomniac patients in the literature [45], there were no significant differences in these consumptions between apneic patients with and without insomnia disorder in this study. This difference from the literature could be explained by the fact that all subjects included in this study were apneic patients with sleep complaints justifying polysomnographic recording regardless of their insomnia complaints. This recruitment, limited only to apneic patients, may potentially have masked the impact of insomnia disorder on alcohol consumption and smoking in this study, since apneic patients are already a subpopulation at a higher risk of alcohol consumption and smoking following their sleep complaints [46,47].

### Limitations and Strengths

Given that the collection of data used was carried out retrospectively without direct verification from the apneic patients included in this study, the performance of additional prospective studies is essential to confirm our findings. Additionally, since only patients with OSAS were recruited for this study, our results cannot be extrapolated to patients with other sleep-related breathing disorders. Furthermore, given that we focused on the potential role played by major depression in the occurrence of comorbid insomnia disorder among apneic patients, the findings of this study cannot be generalized to other psychiatric disorders. Moreover, only apneic patients who have agreed to stay at the Sleep Laboratory for a polysomnography recording are present in the database of the Brussels University Hospital, which may be a limitation regarding the generalization of our results. Finally, despite its limitations, our study is one of the first to investigate the impact of remitted and current major depression (diagnosed during a systematic psychiatric interview) in a large sample of apneic patients, which adds real value compared to the available literature.

## 4. Materials and Methods

### 4.1. Population

**Figure 1:**
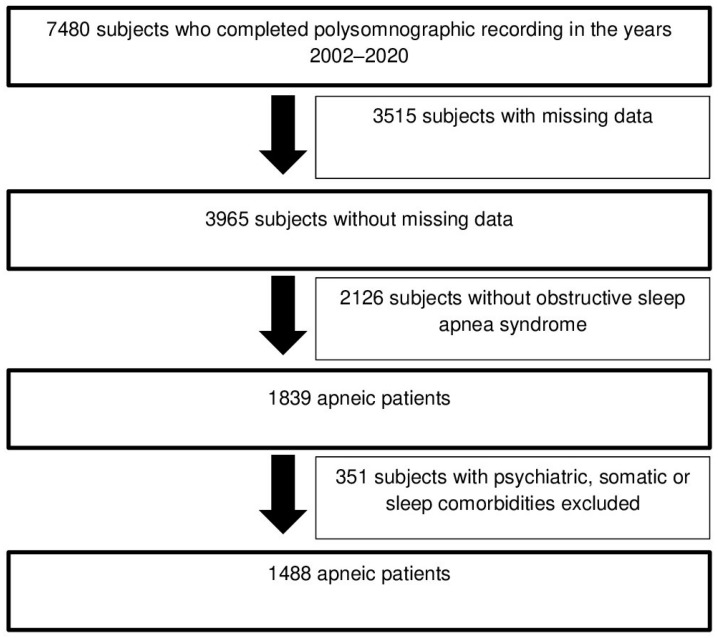
Selection diagram of apneic patients included in this study.

Data from 1488 apneic patients who stayed at the Sleep Laboratory between 1 January 2002 and 31 December 2020 were extracted from the medical reports of polysomnographic recordings available in the database of the Brussels University Hospital (Figure 1). The criteria applied for the selection of these apneic patients are detailed in Table 4 [48]. Furthermore, we decided to focus only on apneic patients for this study, given the potential negative consequences associated with the occurrence of comorbid insomnia disorder in this particular subpopulation [3,4,5,6,7,8]. Finally, the description of the outpatient care journey for these apneic patients, from the specialized consultation for sleep medicine to their admission to the Sleep Laboratory, is detailed in the Supplementary Data—Annex 1 [26].

### 4.2. Method

#### 4.2.1. Medical and Psychiatric Assessment

During their hospitalization for polysomnographic recording, all these apneic patients benefited from a standardized somatic check-up specific to the Sleep Laboratory of the Brussels University Hospital (review of medical records, clinical interview, physical examination and complementary tests [blood test, electrocardiogram, daytime electroencephalogram and urine analyzes]) in order to systematically diagnose their potential medical comorbidities.

Subsequently, a systematic psychiatric interview based on the diagnostic criteria of DSM-IV-TR (before 2013) and DSM 5 (after 2013) was carried out by a psychiatrist assigned to the Sleep Laboratory for all these apneic patients to identify their potential past or current psychiatric comorbidities [37,38]. Thus, following this systematic psychiatric interview, the status of potential major depressive episodes (remitted or current) was determined according to the following criteria:

- The absence of significant symptoms or signs of major depression during a period of at least 2 months before hospitalization for polysomnographic recording was used to define remitted major depressive episodes [37,38].
- The presence of significant symptoms or signs of major depression during a period of at least 2 weeks (DSM 5) or at least 4 weeks (DSM-IV-TR) before hospitalization for polysomnographic recording was used to define current major depressive episodes [37,38].

Finally, after these somatic and psychiatric assessments, all these apneic patients completed a series of questionnaires to determine the severity of their self-reported complaints of depression, insomnia and daytime sleepiness.

- The Beck Depression Inventory (reduced to 13 items) was used to investigate the presence of depressive symptoms. The 13 items of this scale may be scored from 0 to 3, which means that the total score may vary from 0 to 39. A final score of 0–4 indicates an absence of depressive symptoms, 5–7 indicates mild depressive symptoms, 8–15 indicates moderate depressive symptoms, and ≥16 indicates severe depressive symptoms [49]. The internal consistency reliability measure showed a Cronbach α coefficient of 0.90 for the French version of The Beck Depression Inventory (reduced to 13 items) [50].
- The Insomnia Severity Index was used to investigate the severity of insomnia complaints. The 7 items of this index may be scored from 0 to 4, which means that the total score may vary from 0 to 28. A final score of 0–7 indicates an absence of insomnia complaints, 8–14 indicates subclinical insomnia complaints, 15–21 indicates moderate insomnia complaints, and 22–28 indicates severe insomnia complaints [51]. The internal consistency reliability measure showed a Cronbach α coefficient of 0.92 for the French version of the Insomnia Severity Index [52].
- The Epworth Sleepiness Scale was used to investigate daytime sleepiness. The 8 items of this scale assessing sleepiness in different daytime situations may be scored from 0 to 3, which means that the total score may vary from 0 to 24. A final score greater than 10 indicates excessive daytime sleepiness [53]. The internal consistency reliability measure showed a Cronbach α coefficient of 0.88 for the French version of the Epworth Sleepiness Scale [54].

#### 4.2.2. Sleep Evaluation and Study

In all these apneic patients, a systematic interview investigating their sleep habits and their sleep-related complaints was conducted by a psychiatrist specializing in sleep medicine during their hospitalization at the Sleep Laboratory in order to highlight the presence of potential signs suggestive of the main sleep disorders.

Subsequently, to complete this interview focused on sleep, a polysomnographic recording meeting the criteria of the American Academy of Sleep Medicine was carried out in all these apneic patients [55]. The polysomnography instruments applied were as follows: two electro-oculogram channels, three electroencephalogram channels, one submental electromyogram channel, an electrocardiogram, a pressure cannula to detect the oro-nasal airflow, a finger pulse oximeter, a microphone to record breathing sounds and snoring, plethysmographic inductive belts to measure thoracic and abdominal breathing, and anterior tibialis electrodes. Furthermore, the conditions of hospitalization at the Sleep Laboratory for all apneic patients were as follows: (1) the patients went to bed between 22:00–24:00 and got up between 6:00–8:00 (following their usual schedule); (2) during bedtime hours, the subjects were recumbent and the lights were turned off; and (3) daytime naps were not permitted. Finally, a technical report of these polysomnographic recordings was produced by specialized technicians after visual scoring based on the criteria of the American Academy of Sleep Medicine in order to allow their clinical interpretation by physicians specializing in sleep medicine [56,57,58].

Obstructive apneas were scored if the decrease in air flow was ≥90% for at least 10 s whereas obstructive hypopneas were scored if the decrease in airflow was ≥30% for at least 10 s, with a 3% decrease in oxygen saturation or micro-arousal [57]. The obstructive apnea–hypopnea index corresponds to the total number of obstructive apneas and hypopneas divided by the period of sleep in hours [57].

Periodic limb movements were scored based on the following strict criteria: (F1) duration between 0.5 to 10 s; (2) interval between 5 and 90 s from leg movement onset; and 3) movements that were part of a series of ≥4 consecutive movements meeting these criteria [58]. The periodic limb movement index corresponds to the total number of periodic limb movements divided by the period of sleep in hours [58].

The completion of this interview focused on sleep and this polysomnographic recording therefore made it possible to confirm the OSAS diagnosis, to determine the OSAS severity (mild [apnea–hypopnea index ≥ 5/h and <15/h], moderate [apnea–hypopnea index ≥ 15/hour and <30/hour], severe [apnea–hypopnea index ≥ 30/hour]) and to systematically screen for all potential comorbid sleep disorders (insomnia disorder [American Academy of Sleep Medicine Work Group diagnostic criteria], moderate to severe periodic limb movement syndrome [periodic limb movement index ≥ 15/hour], restless legs syndrome [International Restless Legs Syndrome Study Group diagnostic criteria] and short sleep duration [<6 h]) in the apneic patients recruited for this study [59,60,61,62,63].

### 4.3. Statistical Analyses

In order to carry out statistical analyses using Stata 14 software, the 1488 apneic patients were categorized into a subgroup without comorbid insomnia disorder and a subgroup with comorbid insomnia disorder. The presence of comorbid insomnia disorder in these apneic patients was defined based on the diagnostic criteria of the American Academy of Sleep Medicine Work Group [60].

Since the majority of continuous data were distributed asymmetrically (histograms, boxplots and quantile–quantile plots to check the data distribution, and Levene’s test to check the equality of variances), medians with their P25-P75 were used for descriptive analyses and Wilcoxon tests were used for comparison tests. For categorical data, descriptive analyses were carried out using percentages and comparison analyses were carried out using Chi^2^ tests.

The risk of comorbid insomnia disorder (dependent variable) associated with a major depression status (categorized: no, remitted, current) and potential confounding factors (independent variables) was investigated using univariate logistic regression models. After a review of the literature on the risk factors for comorbid insomnia disorder in apneic patients [1,21,64,65,66], the potential confounding factors included in this study were body mass index (categorized: <25 kg/m^2^, ≥25 kg/m^2^), age (categorized: <50 years, ≥50 years), cardiometabolic comorbidities (categorized: 0, 1–2, ≥3), OSAS severity (categorized: mild, moderate, severe), sleep movement disorders (categorized: no, moderate to severe periodic limb movement syndrome, restless legs syndrome alone or combined with periodic limb movements), sleep duration (categorized: <6 h, ≥6 h), and the following binary variables: sex, antidepressant therapy, benzodiazepine receptor agonists, alcohol consumption, smoking and excessive daytime sleepiness. Subsequently, following a hierarchical introduction of the significant confounding factors identified during the univariate analyses, this risk of comorbid insomnia disorder associated with major depression status was adjusted using multivariate logistic regression models.

For the final multivariate logistic regression model, the adequacy was verified by the Hosmer and Lemeshow test, whereas the specificity was verified by the Link test. Additionally, the Wald test and the Nagelkerke R-square were used as additional fit criteria.

Following the conditions of use of multivariate logistic regression analyses (number of subjects per predictor > 10) [67,68], each of the two groups of apneic patients for this study had to contain at least 130 subjects (10 subjects * 13 potential predictors) to ensure the validity of the analyses performed, which was largely achieved in this study.

The results were considered significant when the *p*-value was <0.05.

## 5. Conclusions

Comorbid insomnia disorder was present in 40.9% of apneic patients from our sample, which seems to confirm that this sleep disorder is a frequent comorbidity in this particular subpopulation. In addition, we demonstrated that current and remitted major depression were significantly associated with the occurrence of comorbid insomnia disorder in apneic patients, which seems to indicate that this sleep disorder could be a residual symptom and a marker of major depression in this specific subgroup of patients. Moreover, given the results of this study, the establishment of an adequate treatment for major depressive episodes and their potential residual symptoms seems to be essential for apneic patients to allow the better management of comorbid insomnia disorder and the better prevention of its potential negative consequences in this particular subpopulation. Finally, given the retrospective design of our study, it seems necessary to carry out additional prospective studies to confirm this potential role of major depression in the occurrence of comorbid insomnia disorder in apneic patients thanks to a better level of scientific evidence.

## Figures and Tables

**Table 1:** Polysomnographic data (*n* = 1488).

	Whole Sample (*n* = 1488)	Subjects without Insomnia (*n* = 880)	Subjects with Insomnia (*n* = 608)	*p*-Value
Sleep latency (min)	25.2 (13.0–51.5)	22.5 (12.5–46.0)	29.0 (14.5–60.0)	<0.001
Sleep efficiency (%)	77.9 (67.8–85.1)	78.4 (68.2–85.8)	76.9 (67.2–84.1)	0.017
Sleep period time (min)	452.3 (412.0–485.5)	455.5 (415.3–486.5)	446.0 (406.5–481.8)	0.014
Total sleep time (min)	381.0 (334.0–426.3)	383.0 (336.3–428.0)	375.4 (331.6–424.0)	0.073
% stage 1	8.7 (6.0–12.4)	8.8 (6.3–12.4)	8.5 (5.5–12.3)	0.107
% stage 2	53.8 (47.0–59.8)	54.2 (47.3–60.6)	53.0 (46.5–59.2)	0.088
% stage 3	2.9 (0.2–9.2)	2.8 (0.2–8.5)	3.1 (0.1–10.4)	0.383
% REM sleep	15.7 (11.4–20.1)	15.7 (11.3–20.0)	15.6 (11.4–20.2)	0.815
REM latency (min)	83.0 (59.5–126.7)	83.6 (60.0–124.3)	81.5 (58.8–135.0)	0.644
% WASO	13.2 (7.6–21.4)	13.2 (7.7–21.3)	13.0 (7.4–21.8)	0.799
Number of awakenings	32 (22–48)	33 (22–50)	32 (21–46)	0.131
Micro-arousal index	14 (8–23)	14 (9–24)	13 (8–21)	0.003
Apnoea-hypopnoea index	14 (8–29)	15 (8–33)	13 (7–26)	0.001
Oxygen desaturation index	6 (2–15)	7 (2–17)	5 (1–14)	0.002
Total time under 90% of SaO_2_ (min)	11.5 (1.0–60.5)	13.3 (1.5–61.8)	8.8 (0.5–59.0)	0.043
PLMS index	1 (0–10)	1 (0–10)	2 (0–9)	0.897
	Median (P25–P75)	Median (P25–P75)	Median (P25–P75)	Wilcoxon test

REM = rapid eye movement sleep, WASO = wake after sleep onset, SaO_2_ = oxygen saturation, PLMS = periodic limb movements during sleep.

**Table 2:** Univariate analyses (*n* = 1488).

Variables	Categories	%	Subjects without Insomnia	Subjects with Insomnia	*p*-Value Chi^2^	OR (CI 95%)	*p*-Value
Sex	Female (*n* = 343) male (*n* = 1145)	23.0% 77.0%	19.6% 80.4%	28.1% 71.9%	<0.001	1 0.62 (0.49 to 0.79)	<0.001
Age (years)	<50 (*n* = 708) ≥50 (*n* = 780)	47.6% 52.4%	45.0% 55.0%	51.3% 48.7%	0.016	1 0.78 (0.63 to 0.95)	0.017
BMI (kg/m^2^)	<25 (*n* = 289) ≥25 (*n* = 1199)	19.4% 80.6%	17.6% 82.4%	22.0% 78.0%	0.034	1 0.76 (0.58 to 0.98)	0.034
Antidepressant therapy	No (*n* = 1231) Yes (*n* = 257)	82.7% 17.3%	88.5% 11.5%	74.3% 25.7%	<0.001	1 2.66 (2.02 to 3.51)	<0.001
Benzodiazepine receptor agonists	No (*n* = 1346) Yes (*n* = 142)	90.5% 9.5%	93.8% 6.2%	85.7% 14.3%	<0.001	1 2.50 (1.76 to 3.57)	<0.001
Smoking	No (*n* = 1177) Yes (*n* = 311)	79.1% 20.9%	79.0% 21.0%	79.3% 20.7%	0.889	1 0.98 (0.76 to 1.27)	0.889
Alcohol	No (*n* = 933) Yes (*n* = 555)	62.7% 37.3%	61.4% 38.6%	64.6% 35.4%	0.199	1 0.87 (0.70 to 1.08)	0.199
Cardiometabolic comorbidities	0 (*n* = 305) 1–2 (*n* = 744) ≥3 (*n* = 439)	20.5% 50.0% 29.5%	18.9% 49.3% 31.8%	22.9% 51.0% 26.1%	0.031	1 0.85 (0.65 to 1.12) 0.68 (0.50 to 0.91)	0.032
OSAS severity	Mild (*n* = 772) Moderate (*n* = 347) Severe (*n* = 369)	51.9% 23.3% 24.8%	48.6% 23.5% 27.9%	56.6% 23.0% 20.4%	0.002	1 0.84 (0.65 to 1.09) 0.63 (0.49 to 0.82)	0.002
Sleep movement disorders	No (*n* = 1178) Moderate to severe PLMs alone (*n* = 90) RLS alone or combined with PLMs (*n* = 220)	79.2% 6.0% 14.8%	78.6% 6.4% 15.0%	79.9% 5.6% 14.5%	0.780	1 0.86 (0.56 to 1.34) 0.95 (0.71 to 1.27)	0.780
Sleep duration (hours)	≥6 (*n* = 934) <6 (*n* = 554)	62.8% 37.2%	65.0% 35.0%	59.5% 40.5%	0.032	1 1.26 (1.02 to 1.56)	0.032
EDS	No (*n* = 873) Yes (*n* = 615)	58.7% 41.3%	65.5% 34.5%	48.9% 51.1%	<0.001	1 1.98 (1.61 to 2.45)	<0.001
Major depression status	No (*n* = 886) Remitted (*n* = 247) Current (*n* = 355)	59.5% 16.6% 23.9%	71.4% 15.2% 13.4%	42.4% 18.6% 39.0%	<0.001	1 2.05 (1.54 to 2.74) 4.89 (3.75 to 6.37)	<0.001
Insomnia disorder	No (*n* = 880) Yes (*n* = 608)	59.1% 40.9%					
	Median (P25–P75)				Wilcoxon test		
Age (years)	51 (43–59)		52 (43–60)	50 (42–58)	0.023		
BMI (kg/m^2^)	29.0 (25.8–33.1)		29.0 (26.1–33.2)	28.7 (25.5–33.0)	0.143		
ESS	9 (6–13)		9 (5–12)	11 (7–14)	<0.001		
ISI	13 (8–17)		9 (6–12)	18 (16–21)	<0.001		
BDI	3 (1–7)		2 (1–5)	6 (3–11)	<0.001		

BMI = body mass index, OSAS = obstructive sleep apnea syndrome, PLMs = periodic limb movements during sleep, RLS = restless legs syndrome, EDS = excessive daytime sleepiness, ESS = Epworth sleepiness scale, ISI = insomnia severity index, BDI = Beck depression inventory.

**Table 3:** Multivariate analyses (*n* = 1488).

Variables	Model 1 OR Adjusted (CI 95%)	*p*-Value	Model 2 OR Adjusted (CI 95%)	*p*-Value	Model 3 OR Adjusted (CI 95%)	*p*-Value	Model 4 OR Adjusted (CI 95%)	*p*-Value
Major depression		<0.001		<0.001		<0.001		<0.001
No	1		1		1		1	
Remitted	2.03 (1.52 to 2.72)		1.73 (1.23 to 2.43)		1.73 (1.23 to 2.43)		1.75 (1.24 to 2.47)	
Current	4.67 (3.57 to 6.11)		4.08 (3.03 to 5.47)		4.09 (3.05 to 5.50)		3.68 (2.73 to 4.97)	

Model 1 = Model adjusted for sex, age and BMI. Model 2 = Model adjusted for sex, age, BMI, antidepressant therapy and benzodiazepine receptor agonists. Model 3 = Model adjusted for sex, age, BMI, antidepressant therapy, benzodiazepine receptor agonists and cardiometabolic comorbidities. Model 4 = Model adjusted for sex, age, BMI, antidepressant therapy, benzodiazepine receptor agonists, cardiometabolic comorbidities, OSAS severity, sleep duration and excessive daytime sleepiness. Hosmer and Lemeshow test *p* = 0.798. Link test: linear component *p* < 0.001 and nonlinear component *p* = 0.809. Wald test *p* < 0.001. Nagelkerke R-square = 0.179. BMI = body mass index, OSAS = obstructive sleep apnea syndrome.

**Table 4:** Selection criteria.

Inclusion Criteria	Exclusion Criteria
OSAS according to the diagnostic criteria of the American Academy of Sleep Medicine	Presence of acute and/or uncontrolled somatic, infectious or inflammatory pathologies
Absence of psychiatric disorders other than major depression	Presence of central hypersomnia, parasomnia, predominantly central sleep apnea syndrome or OSAS being treated before hospitalization at the Sleep Laboratory
Absence of substance abuse	Presence of craniofacial or thoracic malformations
Absence of pregnancy	Presence of brain damage or head trauma

OSAS = obstructive sleep apnea syndrome.

## Data Availability

The data presented in this study are available upon request from the corresponding author (the data are not publicly available due to privacy restrictions).

## Supplementary Materials

The following supporting information can be downloaded at: https://www.mdpi.com/article/10.3390/clockssleep6030026/s1, Annex 1: Description of the outpatient care journey for apneic patients from the consultation specialized in sleep medicine to their admission to the Sleep Laboratory.
