# Supplementary material for: Risk of Comorbid Insomnia Disorder Associated with Major Depression in Apneic Patients: A Cross-Sectional Study
Source: Clocks Sleep. 2024 Jul 26;6(3):389–401. doi: 10.3390/clockssleep6030026 (PMC11348371; doi:10.3390/clockssleep6030026)
Supplement: Supplementary file 1 [file clockssleep-06-00026-s001.zip › clockssleep-3036907-supplementary.pdf]

Supplementary Data

Annex 1

Description of the outpatient care journey for apneic patients from the consultation specialized in sleep medicine to their admission to the Sleep Laboratory

These apneic patients were referred to the sleep laboratory by physicians specialized in sleep medicine after an outpatient consultation during which a preliminary assessment of their complaints related to sleep, their ongoing psychotropic/somatic treatments and their somatic/psychiatric comorbidities was systematically performed in order to allow a first diagnostic hypothesis. The polysomnographic examinations were performed in these apneic patients to allow an objective assessment of their sleep complaints and confirm the suspicion of OSAS highlighted during the outpatient assessment.
